# Solid-State Photoluminescence of Diphenylnaphthalenes Studied by Photophysical Measurements and Crystallographic Analysis

**DOI:** 10.3390/molecules29245941

**Published:** 2024-12-16

**Authors:** Minoru Yamaji, Toshiki Mutai, Isao Yoshikawa, Hirohiko Houjou, Hideki Okamoto

**Affiliations:** 1 Division of Molecular Science, Graduate School of Science and Engineering, Gunma University, Ota 373-0057, Gunma, Japan; 2 Department of Materials and Environmental Science, Institute of Industrial Science, The University of Tokyo, Meguro, Tokyo 153-8505, Japan; 3 Environmental Science Center, The University of Tokyo, Bunkyo, Tokyo 113-0033, Japan; 4 Department of Chemistry, Faculty of Environment, Life, Natural Sciences and Technology, Okayama University, Okayama 700-8530, Japan

**Keywords:** solid-state emission, fluorescence quantum yield, fluorescence lifetime, crystal structures, polycyclic aromatic hydrocarbon

## Abstract

Thanks to recent developments in spectrophotometric instruments, the spectra, quantum yields (Φ_f_), and lifetimes (*τ*_f_) of photoluminescence from organic and inorganic compounds can be readily determined not only in solution but also in the solid state. It is known that naphthalene emits fluorescence in solution, but not in the solid state. In a previous paper, we reported that solid-state emission can be seen from biaryl compounds comprised of chromophores that show no emission in the solid state. In this work, we prepared diphenylnaphthalenes (DPNs), and the spectra and the Φ_f_ and *τ*_f_ values of fluorescence were determined in solution and the solid state, as well as the crystallographic features. The 2,6-Diphenylnphthalene (26DPN) showed solid-state emission in the wavelength region for longer than those in solution, while the emission spectra of the others in the solid state were similar to those in solution. The crystal structure of 26DPN belonged to a herringbone motif, whereas those of the others were column-stacked structures. Based on these spectroscopic and crystallographic facts, the relationship between the crystal motif and the emission features in the solid state is discussed.

## 1. Introduction

**Scheme 1:**
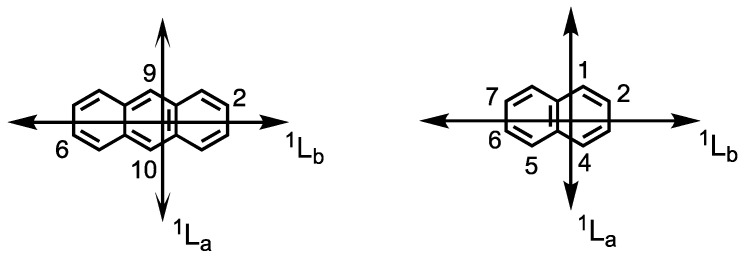
Directions of ^1^L_a_ and ^1^L_b_ transitions and position numbers of the substitution for anthracene (**left**) and naphthalene (**right**) skeletons.

The photophysical properties of polycyclic aromatic hydrocarbons (PAHs) consisting of fused benzene rings closely depend on the number, *n*, of the benzene rings and the molecular structures. PAHs show specific features of absorption and emission in the UV-visible region because of the unique π-electron systems. Photoluminescence has fascinated photochemists, who are eager to understand the photophysical processes and mechanism in solution [1,2,3,4]. Thanks to recent developments in spectrophotometric instruments, the spectra, quantum yields (Φ_f_), and lifetimes (*τ*_f_) of photoluminescence from organic and organometallic compounds can be readily determined not only in solution but also in the solid state [5]. Generally, no emissions from most of the typical PAHs, such as naphthalene, phenanthrene, and pyrene, are observable in the solid state. Anthracene is one of exceptions concerning solid-state emission (Φ_f_ = 0.64 [6]), whereas the fluorescence appears in the blue-color wavelength region in solution with Φ_f_ = 0.27 [7]. Substitution with phenyl functionality on an anthracene core has been one of strategies used for increasing fluorescence efficiencies in solution. In fact, 9,10-diphenylanthracene shows intense fluorescence with Φ_f_ = 0.97 in solution [5], but the Φ_f_ value for the solid-state fluorescence is as small as 0.18 [8]. By contrast, 2,6-diphenylanthracene has moderate Φ_f_ = 0.49 in THF [9] and 0.41–0.48 in the solid state [10]. The difference in Φ_f_ values in solution between these diphenylanthracenes may be derived from the substituting positions by considering the directions of the transient moments (Scheme 1) [1,4].

When the positions on the direction of the allowed transition moment, ^1^L_a_, along the short axis of the chromophore core are occupied on substitution, the fluorescence rate is generally enhanced, resulting in increasing the efficiency of the fluorescence. Vice versa, with the forbidden transition, ^1^L_b_, along the long axis, the fluorescence rate will be less intensified. Phenanthrene, the structural isomer of anthracene, shows substantially weaker fluorescence, with Φ_f_ = 0.05 [11,12]. It is reported that modifying phenanthrene by substituting with fewer phenyl groups causes the Φ_f_ values to be as small as 0.1 [12].

Conversely, the photophysical properties of naphthalene, a smaller homologue of anthracene, have been fully revealed. Naphthalene shows fluorescence in solution with Φ_f_ = 0.19 [13], but not in the solid state. Similarly, phenanthrene and pyrene show no solid-state emission. In a previous paper, we reported that solid-state emission in the visible wavelength region is observable from biaryls composed of these non-solid-state-emissive chromophores [14]. Interestingly, it was found that the biaryl compounds show intense fluorescence in solution more than the parent chromophore. Based on the experimental finding that biaryl molecules with herringbone structures show red-shifted emission spectra in the solid state compared with those in solution, the correlation between the motifs of the crystal structures and the emission spectra in the solid state was investigated. The relationship between the crystal motifs and the shifts in the emission spectra were verified by using the multichromophoric compounds that were expected to emit the solid-state emission.

**Scheme 2:**
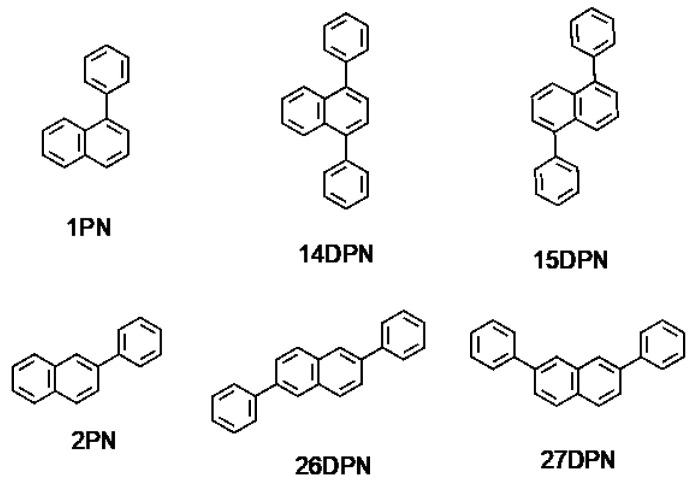
Molecular structures and abbreviations of phenylnaphthalenes (**PNs**) and diphenylnaphthalenes (**DPNs**) studied in this work.

In this context, we have been involved in revealing the relationship between the photophysical features of emissive molecules in the solid state and the crystal structures [15,16,17]. To our knowledge, however, few PAHs are known to show efficient solid-state photoluminescence. Therefore, in the present work, we prepared phenylnaphthalenes (PNs) and diphenylnaphthalenes (DPNs) substituted at various positions (see Scheme 2 for the chemical structures), and studied the solid-state photoluminescence. We determined the Φ_f_ and *τ*_f_ values in solution and the solid state, and analyzed the crystal structures. Based on these data, the relationship between the photophysical features in the solid state and the crystal structures is discussed.

## 2. Results and Discussion

### 2.1. X-Ray Crystallographic Analysis of Crystals

First, the crystal structures of the studied DPNs were analyzed by X-ray diffraction. The obtained crystal features and ORTEP of the DPNs, except for 26DNP, are presented in the Supplementary Materials (SMs) as Figures S6–S9. Pictures of the actually prepared crystals are presented in Figure S10 in the SMs. The crystallographic data for the DPNs are shown in Tables S1 and S2 in the Supplementary Materials, whereas the geometrical parameters for the DPNs, i.e., the twist angles between the naphthalene core and the phenyl rings, and the short contacts found with Mercury [18], are listed in Tables S3 and S4 in the Supplementary Materials, respectively.

The crystal structure of the 14DPN shown in Figure S2 was similar to one that was previously reported (CCDC 802922 [19]), in which two molecules were found in the asymmetric unit. The 14DPN molecules seem to pile up along the *b*-axis to form stacked columns. The naphthalene moieties aligned in a parallel orientation to form a slipped-stack arrangement. However, the π-π interaction would be negligible because the distance between the naphthalene moieties is too long (ca. 4.70 Å, see Figure S6c,d) for each naphthalene chromophore to interact. Additionally, several CH-π contacts between the columns stabilize the crystal structure (Figure S6b and Table S4).

The 15DPN was crystallized as a mixture of needle and plate crystals. These crystals were isostructural to each other. Similar to the case of the 14DPN, the 15DPN molecules formed columnar stacking structure along the *a*-axis (Figure S7b), and the naphthalene moieties were in a parallel, slipped-stack arrangement. Only the edges of the naphthalene moieties overlapped with the distance of 3.41 Å (Figure S7d). The naphthalene–naphthalene contact distance was in a good agreement with a typical π-π staking structure (3.3–3.8 Å [20]), while no other intermolecular interaction was found. 

**Figure 1:**
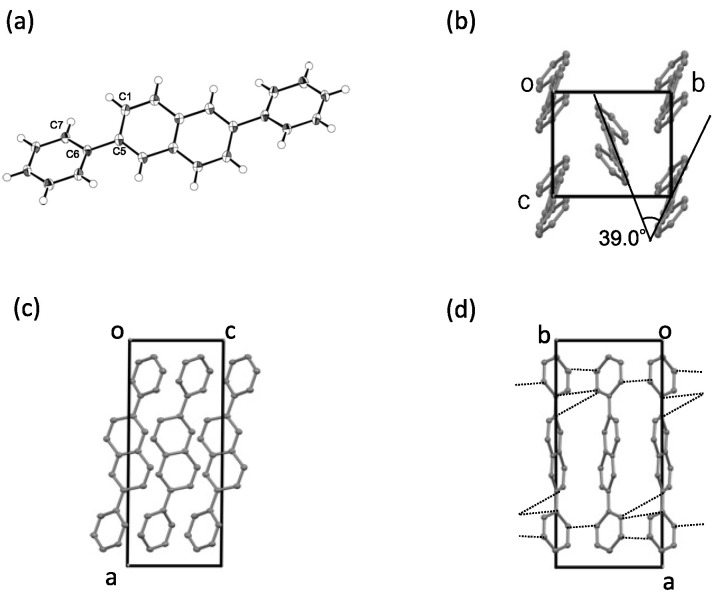
Crystal features of 26DPN. (**a**) ORTEP for the molecular unit. (**b**) View along the *a*-axis. The dihedral angle between the naphthalene rings was 39.0°. (**c**) View along the *b*-axis. (**d**) View along the *c*-axis. The dotted lines denote intermolecular CH-π contacts between the chromophores.

The 26DPN was crystallized as a mixture of prism and plate crystals, but assembled in a thoroughly isomorphous manner. Figure 1 shows the crystal structure of 26DPN. The crystal structure was assembled into two-dimensional layers, in which the naphthalene and phenyl moieties aligned to form two-dimensional layer structures. The naphthalene moieties were thus aligned in a herringbone structure. The distance and angle between these naphthalene rings were 4.86 Å and 39.0°, respectively. No specific intermolecular interaction was found between the naphthalene moieties, probably because the angle between these naphthalene moieties was shallow (39.0°). The phenyl rings were orthogonally arranged to each other (86.1°). The CH-π contact lengths are listed in Table S4. 

Similarly to the case of the 26DPN, the 27DPN molecules also assembled two-dimensionally into a layered structure with the naphthalene and phenyl moieties. Two polymorphs were obtained for the 27DPN crystals, which consisted of major, nearly square plate crystals, with a very small amount of orthogonal plate crystals. The crystal systems of the major and minor components were monoclinic *C*c and orthorhombic *P*nma, which were named as 27DPN-m and 27DPN-o, respectively. The asymmetric unit of 27DPN-m contains four molecules, in which the torsion angles between the naphthalene and phenyl moieties are slightly different (Table S3). The naphthalene moieties are orthogonally arranged and connected with double CH-π contacts between the 1, 8 to 4, 5 positions and the 4, 5 to 1, 8 positions. Additionally, various CH-π contacts from the phenyl to the phenyl and naphthalene were observed (Figure S8, Table S4). The crystal structure of the minor form, 27DPN-o, seemed to be similar to that of the 26DPN (Figure S9). No special intermolecular interaction was found between the naphthalene moieties. Three CH-π contact distances between one phenyl and the other phenyl connecting to the naphthalene moiety in the neighbor are listed in Table S4.

### 2.2. Spectroscopic Features

**Figure 2:**
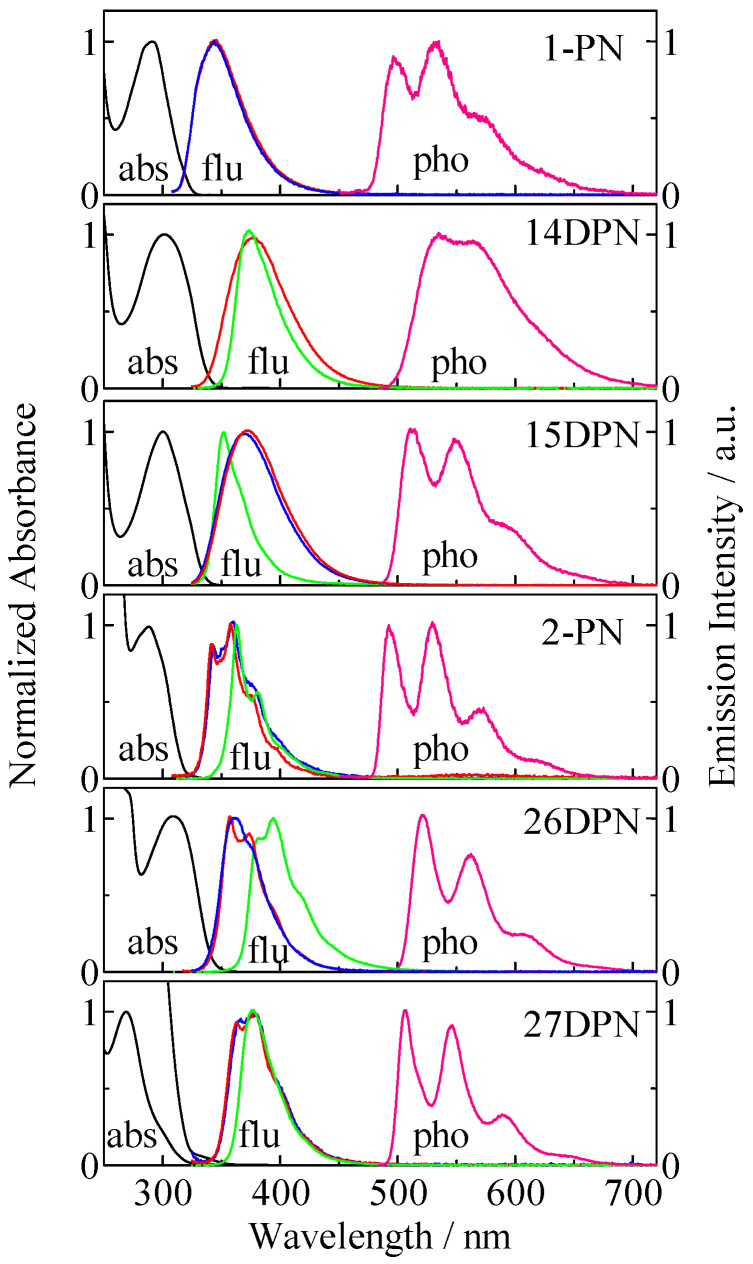
Absorption (black) and fluorescence spectra in cyclohexane (blue) and acetonitrile (red) at 295 K, fluorescence spectra (green) in the solid state at 295 K, and phosphorescence spectra (magenta) in ethanol at 77 K for the studied PNs and DPNs. The emission spectra are not corrected.

Figure 2 shows the absorption and emission spectra of the PNs and DPNs studied in the present work.

From the studied DPs and DPNs, fluorescence was observed in solution in the deep-blue wavelength region (340–440 nm). Solid-state emission was also observed from the studied compounds, except for 1-PN, as it is liquid at room temperature. The fluorescence spectra of the 14- and 15DPNs had no vibrational structures, as was seen for the 1-PN, whereas those of the 26- and 27DPNs clearly showed these structures, as was observed with the 2PN. The 14-, 15-, and 27DPNs showed their emission spectra in solution in a similar wavelength region to those in the solid state, whereas the 26DPN showed fluorescence in the solid state in a different wavelength region from that in the solution. The fluorescence spectra in acetonitrile were similar to those in cyclohexane, indicating that the fluorescent states of DPNs possess little charge-transfer character. The observation of the phosphorescence indicated that an intersystem crossing from the S_1_ state to the triplet is operative in the studied compounds. The characteristic wavelength of fluorescence (λ_f_) at the maxima for the 14- and 15DPNs and at the 0-0 band for the 26- and 27DPNs are listed in Table 1.

It should be noted that solid-state emission from the studied compounds, except 1-PN, was observable, and that the Φ_f_ values were moderately large, in contrast to the lack of solid-state emissions from the naphthalene. This fact suggests that solid-state emission from PAHs can be induced by multi-chromophorization. As with fluorescence in solution, the Φ_f_ values of the substituted naphthalenes at the α-position on the naphthalene skeleton (1-PN, 1,4-, and 1,5DNP) were greater than that of naphthalene (0.19 [21]), whereas the 2-PN, 2,6-, and 2,7DPNs substituted at the β-position showed lower Φ_f_ values. The rates for the fluorescence (*k*_f_) and nonradiative (*k*_nr_) processes were determined by Equations (1) and (2), respectively, and listed in Table 1.

*k*_f_ = Φ_f_*τ*_f_^−1^, (1)

*k*_nr_ = (1−Φ_f_)*τ*_f_, (2)

The rates, *k*_f_ and *k*_nr_, determined in solution and the solid state were crucial to evaluate the photophysical features of the studied DPNs. Independently of the environment (solution and the solid state), the *k*_f_ values of the 14- and 15DPNs that were substituted with phenyl groups along the direction of the ^1^L_a_ transition were greater (10^8^ s^−1^) by one or two orders compared to those (10^6^–10^7^ s^−1^) for the 26- and 27DPNs that had the phenyl groups along the direction of the ^1^L_b_ transition. These results are reasonable because the ^1^L_a_ and ^1^L_b_ have the character of allowed and forbidden transitions, respectively. The enhancement in the fluorescence process by introducing phenyl groups along the direction of the ^1^L_a_ transition resembles the case of 9,10-diphenylanthracene [1,4]. The magnitude of the determined *k*_nr_ values is similar to that of the *k*_f_ values. Assuming that the Ermolaev rule is applicable to PNs and DPNs, the *k*_nr_ values would be for intersystem crossing from the S_1_ state to the triplet state. The triplet energies, *E*_T_, determined from the phosphorescence origins are tabulated in Table 1. The *E*_T_ values of the DPNs are similar to each other, and they are smaller than the *E*_T_ values of the PNs.

The emission spectra seen in solution and the solid-state emission were mirror-imaged to the corresponding absorption spectra. As with the substituted naphthalenes at the β-position on the naphthalene skeleton (2-PN, 2,6-, and 2,7DNP), the vibrational structures were seen in the emission spectra in solution and in the solid state. These observations indicate that the obtained emission originated from the local excited state of the DPNs, not from excimeric and intermolecular π-π* interactions between the neighboring phenyl and naphthyl chromophores. The crystal motif of the 26DPN is of a herringbone, whereas those of the 14-, 15-, and 27DPNs belonged to column-stacked motifs. The emission spectra of the 14-, 15-, and 27DPNs in solution were similar to those in the solid state, whereas that of the 26DPN in the solid state appears in the longer wavelength region compared to that in solution. These experimental results agree with the prediction obtained from the previous studies on photophysics and the crystallography of biaryls [14], i.e., biaryl compounds having a herringbone structure as the single crystal motif show the solid-state emission appearing in a lower energy region than that in solution. Consequently, we can generalize the relationship between the emission spectral shift and the crystal motif for PAH multi-chromophore compounds. That is, multi-chromophore compounds having herringbone structures show solid-state emission red-shifted to that in solution. Vice versa, once solid-state emission red-shifted from that in solution is observed, the crystal structure can be predicted to belong to a herringbone motif.

To verify whether this relationship between solid-state emission and crystal structures is applicable to PAHs other than DPNs, we examined the reported data of 2,6-diphenylanthracene (26DPA) [10]. The emission spectrum of the 26-DPA showing vibrational structures in the solid state definitely appeared in the energy region lower than that in the solution, and the crystal structure was of a herringbone. The data for the 26DPA follows the relationship between the emission spectral shift and the crystal motif. Similarly, our unpublished emission spectrum in the solid state of 9,10-anthracene shows the red-shift compared to that in the solution (see Figure S1 in Supplementary Materials), whereas the crystal structure is reported to be of a herringbone [22,23].

### 2.3. Quantum Chemical Calculation

To obtain deeper insight into the photophysical properties of the studied DPNs, DFT and TD-DFT calculations were carried out at the (TD)-B3LYP/6-31+G(d) level considering cyclohexane as the solvent [24]. The calculation results are summarized in Table 2. 

**Figure 3:**
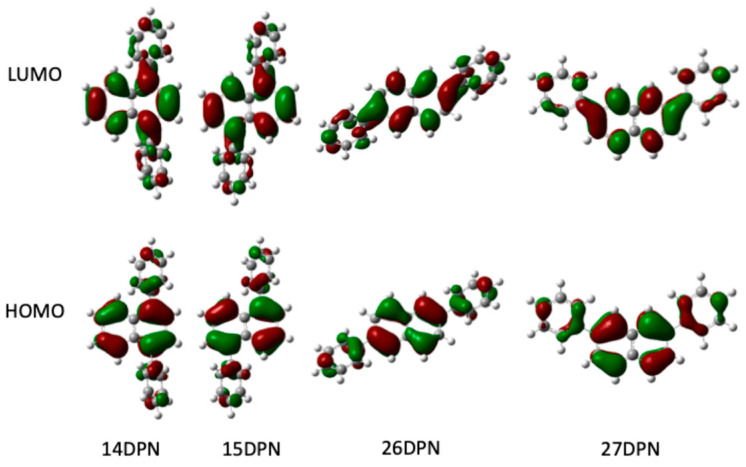
The HOMO and LUMO surfaces of the studied DPNs in cyclohexane.

The calculated molecular orbital diagrams, the highest occupied molecular orbitals (HOMO), and the lowest unoccupied molecular orbitals (LUMO) in vacuum are shown in Figure 3.

In the optimized structures of the DPNs, the HOMO and the LUMO are located over the chromophores. The S_1_ ← S_0_ transitions of the 14- and 15DNPs are solely contributed by the HOMO (H) → LUMO (L) configuration, whereas those of the others consist of various transitions among the occupied and vacant MOs. The wavelengths (*λ*_tr_) estimated from the transition energies are in the 320–330 nm wavelength region. The magnitude of the calculated oscillator strengths (*f*) for the S_1_ ← S_0_ transitions for the DPNs, except for 27DPN, is responsible for the allowed transition, whereas the calculated result of *f* = 0 for 27DPN indicates that the S_1_ ← S_0_ transition is forbidden. The fluorescence rate, *k*_f_, is related to the oscillator strength, *f*, via the Strickler–Berg equation [25]. In fact, the determined *k*_f_ values of the 27DPN in solution and in the solid state are one or two orders smaller than those of the others.

## 3. Experimental

The studied DPNs were prepared by the procedures described in Supplementary Materials (SM). Cyclohexane, acetonitrile, and ethanol (spectroscopic grade from Wako) were used as solvent for the spectral measurements without further purification. NMR spectra for DPNs recorded on a JEOL JNM-ECZ600R, Tokyo, Japan (600 MHz) spectrometer are presented in Figures S2–S5 in Supplementary Materials. The high-resolution fast atom bombardment mass spectra (HR-FAB-MS) were obtained with 3-nitrobenzyl alcohol as a matrix, and recorded on a double-focusing magnetic sector mass spectrometer (JEOL JMS-700, Tokyo, Japan). Absorption spectra in solution were recorded on a JASCO V-730 spectrophotometer, Tokyo, Japan. Fluorescence quantum yields (Φ_f_) and fluorescence spectra in solution and the solid state were obtained with an absolute photoluminescence quantum yield measurement system (C13534-01) from HAMAMATSU PHOTONICS K.K., Hamamatsu, Japan, upon photoexcitation at 315 nm, whereas fluorescence lifetimes (*τ*_f_) in solution and the solid state were determined with a time-correlated single-photon counting fluorimeter system (C16361-01) from HAMAMATSU PHOTONICS K.K. Excitation wavelengths on measuring emission lifetimes are noted in SM. Samples in a quartz cuvette with a 1 cm optical path length were subjected to measurements for Φ_f_ and *τ*_f_ in aerated solution. Single crystals of DPNs were prepared from a mixture of *n*-hexane and chloroform, typically from chloroform solution, to which hexane was gradually added until the mixture narrowly started sedimentation. Single-crystal X-ray diffraction data were collected on a Rigaku XtaLAB P200, Tokyo, Japan, *λ* (Cu-Kα) = 1.54184 Å. The structure was solved by direct method (SHELXS-2013 [26]) and refined on *F*^2^ by full-matrix least-squares techniques (SHELXL-2018 [27]), controlled by the Yadokari-XG software package, Japan [28]. The crystal of 27PNP-m (see the following section for details) was twinned, and the twinning was resolved by TwinRotMat command in the PLATON [29]. The theoretical calculations for the studied DPNs were carried out at the DFT level, using the Gaussian 09 software package [24]. The geometries of the studied compounds were fully optimized at the 6-31G(d)/B3LYP level based on the self-consistent reaction field (SCRF) theory using conductor-like polarizable continuum model (CPCM) considering the dielectric constant of cyclohexane. The atom coordinates for the optimized geometries are presented in Supplementary Materials. Time-dependence DFT (TD-DFT) calculations were performed at TD B3LYP/6-31+G(d) level using the optimized geometries.

## 4. Conclusions

As we observed solid-state emission from biaryl compounds in our previous research [14], we proposed that PAH compounds comprising multichromophores would give solid-state emissions. Some biaryls showed red-shifted spectra of the solid-state emission compared to the emission spectra in the solution. Furthermore, the single-crystal structures belonged to herringbone motifs. Based on these previous results of crystallography and emission features, we paid attention to the relationship between crystal structures and emission spectra features. In the present study, we prepared DPNs and analyzed the crystal structures. A herringbone structure was found for 26DPN, while the other DPNs had stacked column structures. Photoluminescence in the solid state, as well as in solution, was observed, and the Φ_f_ and *τ*_f_ values were determined. Based on these photophysical parameters, the substituent position effect on the photophysical properties of the DPNs was interpreted in terms of the direction of the transition moment in the excited singlet states. The fluorescence of 26DPN in the solid state appeared in the wavelength regions for longer than that in solution, while the solid-state emissions of the other DPNs appeared in the same wavelength region as the fluorescence in solution. The present results confirm the proposed relationship between the solid-state emission and the crystal structures. This relationship would be useful in screening PAH molecules for electronic semiconductor materials because electronically functional molecules are apt to possess herringbone crystal structures [30,31]. For example, 26DPA, whose crystal structure is that of a herringbone [10], follows this relationship, and it is reported to behave as a semiconductor [10,32,33,34,35]. The semiconducting properties of 26DPN will need to be verified in the future.

## Figures and Tables

**Table 1:** Photophysical parameters in aerated cyclohexane at 295 K for PN and DPNs studied in the present work ^1^.

Compound	λ_f_ /nm	Φ_f_	*τ*_f_^2^ /ns	*k*_f_ ^3^ /10^7^ s^−1^	*k*_nr_ ^4^ /10^7^ s^−1^	*E*_T_ ^5^ /kcal mol^−1^
1-PN ^6^	348 (345)	0.20 (0.18)	7.5 (5.8)	2.7 (3.1)	10.7 (14.1)	57.4
2-PN	342 (342) [364]	0.05 (0.07) [0.53]	17.1 (13.7) [76.6]	0.3 (0.5) [0.69]	5.6 (6.8) [0.61]	58.0
14DPN	378 (376) [372]	0.42 (0.38) [0.58]	1.0 (1.0) [1.9]	42.0 (38.0) [30.5]	58.0 (62.0) [22.1]	53.6
15DPN	370 (371) [351]	0.57 (0.46) [0.34]	1.7 (1.7) [7.1]	33.5 (27.1) [4.8]	25.3 (31.8) [9.3]	55.8
26DPN	358 (358) [414]	0.13 (0.15) [0.76]	13.6 (32.0) [12.0]	1.0 (0.5) [6.3]	6.4 (2.7) [2.0]	54.7
27DPN	365 (365) [376]	0.07 (0.11) [0.39]	18.8 (15.3) [97.5]	0.4 (0.7) [0.4]	4.9 (5.8) [0.6]	56.3

^1^ Data in parentheses and brackets are for the acetonitrile and the solid state, respectively. ^2^ Excitation wavelengths are noted in SMs. ^3^ Evaluated by Equation (1). ^4^ Evaluated by Equation (2). ^5^ Determined from the phosphorescence origin. ^6^ As 1-PN is liquid at room temperature, the solid-state emission is out of the scope of this research.

**Table 2:** Calculated photophysical parameters in cyclohexane for the studied DPNs.

Compound	HOMO/eV	LUMO/eV	λ_tr_ ^1^/nm	*f* ^2^	Coefficient for the S_1_ ← S_0_ Transition ^3^
14DPN	−5.89	−1.57	323	0.4769	0.69938 (H → L)
15DPN	−5.88	−1.47	320	0.4456	0.69874 (H → L)
26DPN	−5.84	−1.61	322	0.5980	0.69577 (H → L)
27DPN	−5.83	−1.71	328	0.0002	−0.49016 (H-1 → L) 0.49522 (H → L + 1)

^1^ Wavelength estimated from the transition energy. ^2^ Oscillator strength for the S_1_ ← S_0_ transition. ^3^ H and L denote HOMO and LUMO, respectively.

## Data Availability

The original contributions presented in the study are included in the article/Supplementary Materials, and further inquiries can be directed to the corresponding author.

## Supplementary Materials

The following supporting information can be downloaded at https://www.mdpi.com/article/10.3390/molecules29245941/s1. Figure S1. Absorption and fluorescence spectra of 9,10-diphenylanthracene in cyclohexane and the solid state, Figure S2. ^1^H (600 MHz, CDCl_3_) and ^13^C (151 MHz, CDCl_3_) NMR spectra of 14DPN, Figure S3. ^1^H (600 MHz, CDCl_3_) and ^13^C (151 MHz, CDCl_3_) NMR spectra of 15DPN, Figure S4. ^1^H (600 MHz, CDCl_3_) and ^13^C (151 MHz, CDCl_3_) NMR spectra of 26DPN, Figure S5. ^1^H (600 MHz, CDCl_3_) and ^13^C (151 MHz, CDCl_3_) NMR spectra of 27DPN, Figure S6. Crystal features of 14DPN, Figure S7. Crystal features of 15DPN, Figure S8. Crystal features of 27DPN-m, Figure S9. Crystal features of 27DPN-o, Table S1. Crystallographic data of 14DPN and 15DPN, Table S2. Crystallographic data of 26DPN and 27DPN, Table S3. Torsion angles of the phenyl groups, Table S4. Short contact between D and A found in Mercury software Ver 4.3.1, Figure S10. Pictures of the prepared crystals, Figure S11. Decay profiles of fluorescence for PNs and DPNs in cyclohexane at 295 K, Figure S12. Decay profiles of fluorescence for PNs and DPNs in acetonitrile at 295 K, Figure S13. Decay profiles of fluorescence for 2PN and DPNs in the solid state at 295 K, Table S5. Atom coordinates for the optimized geometry of 14DPN, Table S6. Atom coordinates for the optimized geometry of 15DPN, Table S7. Atom coordinates for the optimized geometry of 26DPN, Table S8. Atom coordinates for the optimized geometry of 27DPN. References [19,24,36] are cited in the supplementary materials.
