# Supplementary material for: Solid-State Photoluminescence of Diphenylnaphthalenes Studied by Photophysical Measurements and Crystallographic Analysis
Source: Molecules. 2024 Dec 16;29(24):5941. doi: 10.3390/molecules29245941 (PMC11677213; doi:10.3390/molecules29245941)
Supplement: Supplementary file 1 [file molecules-29-05941-s001.zip › molecules-3298026-supplementary.pdf]

Supplementary Materials for

Solid-state photoluminescence of diphenylnaphthalenes studied by photophysical measurements and crystallographic analysis

Minoru Yamaji,[a,*], Toshiki Mutai,[b] Isao Yoshikawa,[b] Hirohiko Houjou,[b,c] and Hideki Okamoto[d]

[a] Division of Molecular Science, Graduate School of Science and Engineering, Gunma University, Ota, Gunma 373-0057, Japan
[b] Department of Materials and Environmental Science, Institute of Industrial Science, The University of Tokyo, Meguro, Tokyo 153-8505, Japan
[c] Environmental Science Center, The University of Tokyo, Bunkyo, Tokyo 113-0033, Japan
[d] Department of Chemistry, Faculty of Environment, Life, Natural Sciences and Technology, Okayama University, Okayama 700-8530, Japan

Contents

P. 2 **1. Unpublished photophysical data for 9,10-diphenylanthracene. (Figure S1)**

P. 3 **2. Materials.**

P. 4 **3. ^{1}H and ^{13}C NMR spectra of the synthesized DPNs. (Figure S2-5)**

P. 8 **4. X-ray crystallographic analysis data of DPNs. (Figure S6-10, Tables S1-4)**

P. 16 **5. Decay profiles of fluorescence of the DPNs in cyclohexane, acetonitrile and the solid state. (Figure S11-13)**

P. 19 **6. Results of DFT and TD-DFT calculations of the DPNs; atom coordinates and sum of electronic and zero-point energies in cyclohexane. (Tables S5-8)**

P. 24 **References**

1. Unpublished photophysical data for 9,10-diphenylanthracene

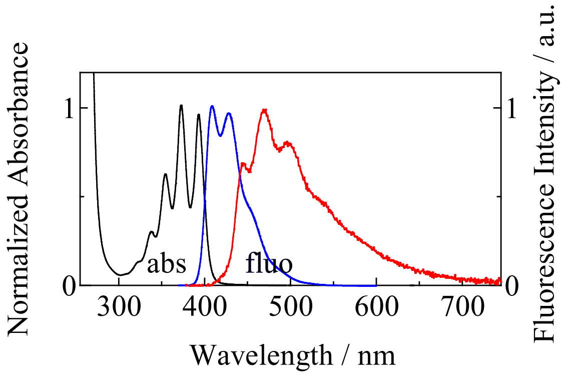

Figure S1. Absorption (solid) and fluorescence spectra of 9,10-diphenylanthracene in cyclohexane (blue) and the solid state (red).

2. Materials

2-1. Chemicals

Cyclohexane and acetonitrile (spectroscopic grade from Wako) were used as solvents for the spectral measurements as supplied. The studied DPNs were prepared by the procedures described blows.

2-2. General synthesis of DPNs.

To a mixture of dimethoxyethane (10 ml) and H_2O (2 ml), dibromonaphthalene (1.0 mmol), phenyl boronic acid (2.4 mmol) and K_2CO_3 (690 mg, 5.0 mmol) were added, and the solution was bubbled with pure N_2 for 10 min. After tetra(triphenylphosphinate)palladium (0) (120 mg, 10 % mol) was added, the solution was heated at 85 °C for 4-22 h. After cooling to room temperature, benzene (100 ml) was added. The solution was washed twice with brine (100 ml). Then, the organic layer was separated, and dried with Na_2SO_4. After the organic solvent was evaporated, the crude product was separated by silica gel chromatography with a hexane/chloroform mixture (3:1, v/v) as the eluent, and purified by recrystallization from hexane.

2-3. Analytical data for the synthesized DPNs.

1,4-Diphenylnaphthalene; **14DPN**: Heating duration 12 h. Yield 92 %. ^{1}H NMR (600 MHz, $CDCl_3$) δ_H 7.98 (AA'BB', 2H), 7.57-7.50 (m, 8H), 7.48 (s, 2H), 7.47-7.42, m, 4H).^{13}C NMR (151 MHz, $CDCl_3$) δ_C 140.96, 139.97, 132.06, 130.29, 128.44, 127.42, 126.60, 126.53, 125.99. The obtained data was identical with those reported previously.[1] HRMS (FAB) *m/z* calcd. for $C_{22}H_{16}$ 280.1252, found 280.1252.

1,5-Diphenylnaphthalene; **15DPN**: Heating duration 4 h. Yield 21 %.^{1}H NMR (600 MHz, $CDCl_3$) δ_H.7.91 (dd, 2H, J = 7.8, 1.1 Hz), 7.55−7.49 (m, 8H), 7.49−7.42 (m, 6H). ^{13}C NMR (151 MHz, $CDCl_3$) δ_C. 141.20, 140.68, 132.13, 130.29, 128.41, 127.40, 127.04, 125.90, 125.53. HRMS (FAB) *m/z* calcd. for $C_{22}H_{16}$ 280.1252, found 280.1252.

2,6-Diphenylnaphthalene; **26DPN**: Heating duration 22 h. Yield 45 %.^{1}H NMR (600 MHz, $CDCl_3$) δ_H 8.07 (d, 2H, J = 1.8 Hz), 7.98 (d, 2H, J = 8.4 Hz), 7.79 (dd, 2H, J = 8.2, 1.5 Hz), 7.77-7.73 (m, 4H), 7.53-7.48 (m, 4H), 7.41-7.38 (m, 2H).

^{13}C NMR (151 MHz, $CDCl_3$) δ_C 141.17, 138.74, 132.97, 129.03, 128.86, 127.53, 126.17, 125.66. HRMS (FAB) *m/z* calcd. for $C_{22}H_{16}$ 280.1252, found 280.1252.

2,7-Diphenylnaphthalene; **27DPN:** Heating duration 11 h. Yield 79 %. ^{1}H NMR (600 MHz, $CDCl_3$) δ_H 8.10 (m, 2H), 7.94 (m, 6H), 7.50 (m, 4H), 7.39 (m, 2H). ^{13}C NMR (151 MHz, $CDCl_3$) δ_C 141.23, 139.16, 134.08, 131.92, 129.02 (two lines overlap), 128.31, 127.57, 127.56, 126.23, 125.86. HRMS (FAB) *m/z* calcd. for $C_{22}H_{16}$ 280.1252, found 280.1252.

3. ^{1}H and ^{13}C NMR spectra of the synthesized DPNs.

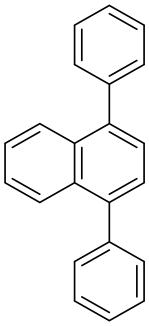

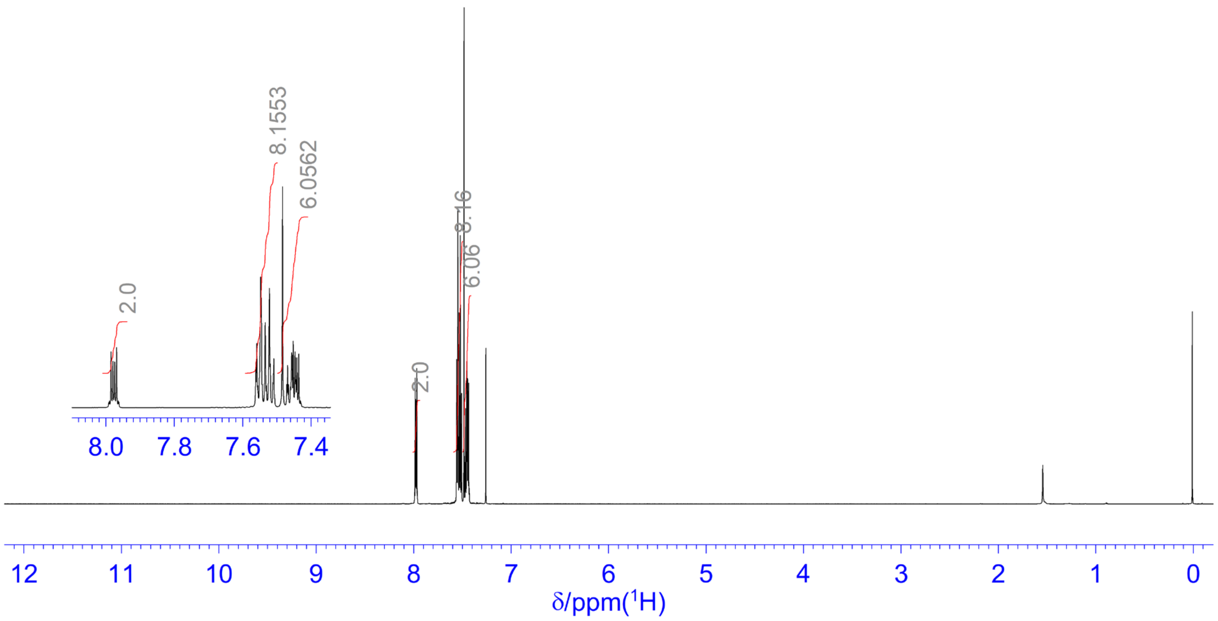

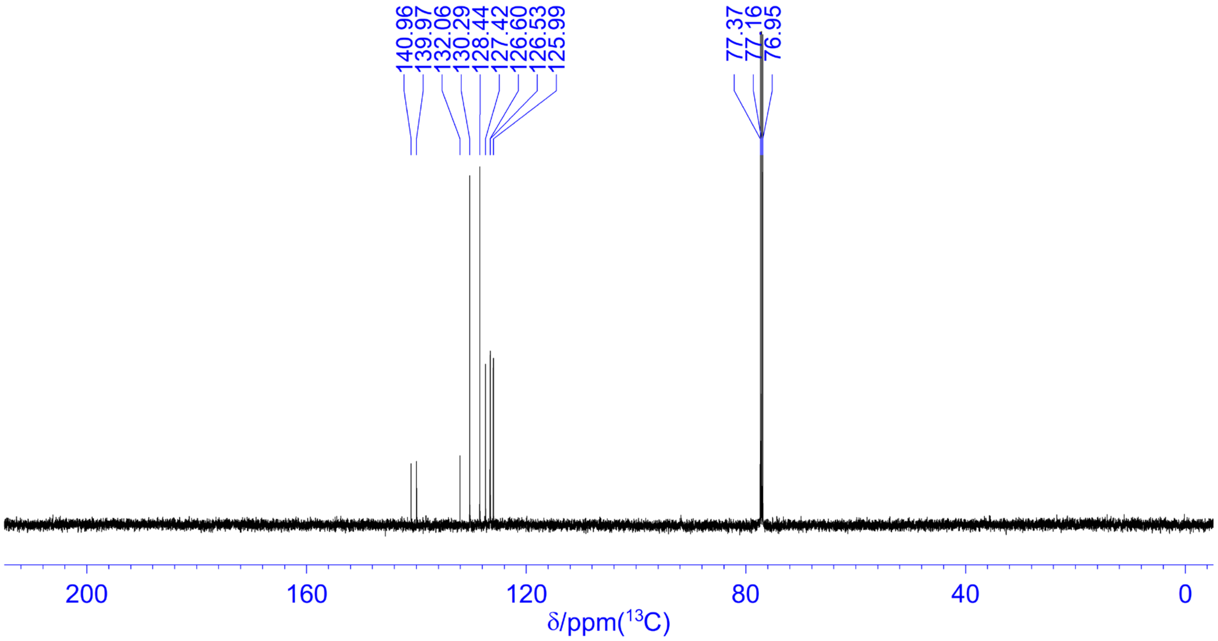

Figure S2. ^{1}H (600 MHz, $CDCl_3$, upper) and ^{13}C (151 MHz, $CDCl_3$, lower) NMR spectra of **14DPN**.

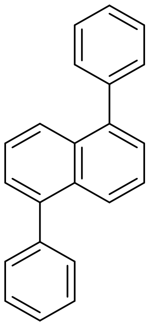

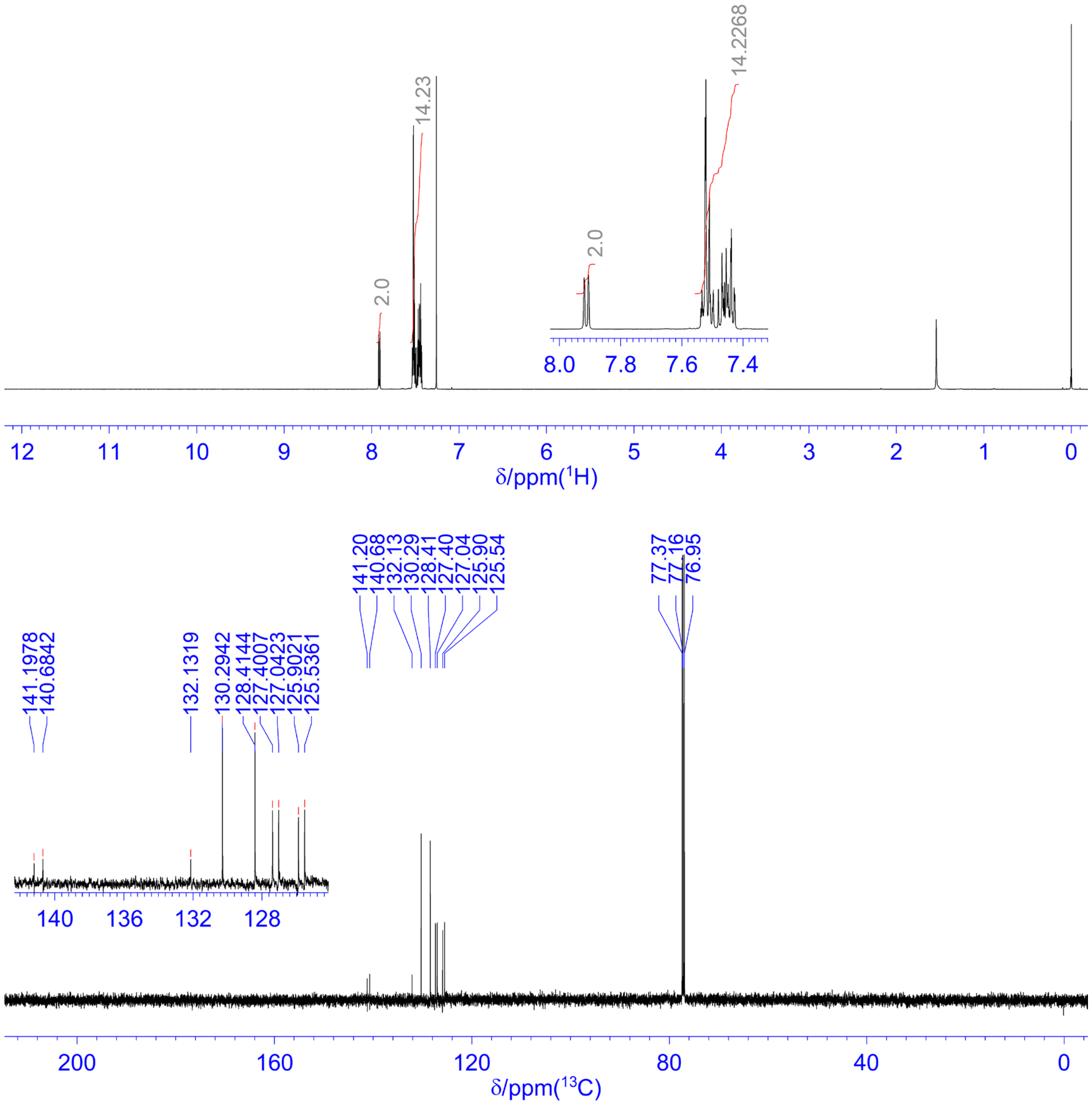

Figure S3. ^{1}H (600 MHz, $CDCl_3$, upper) and ^{13}C (151 MHz, $CDCl_3$, lower) NMR spectrum of **15DPN**.

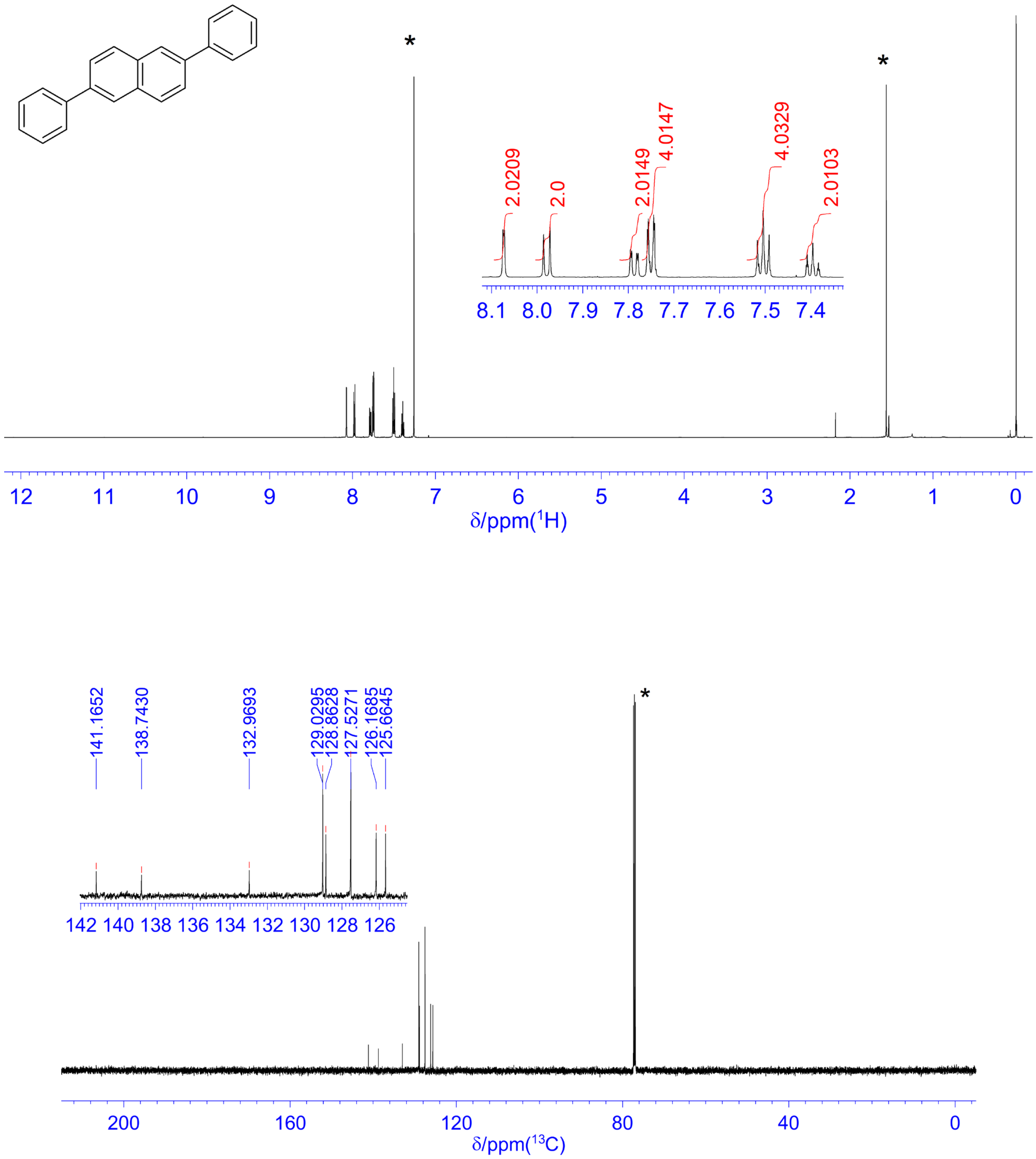

Figure S4. ^{1}H (600 MHz, $CDCl_3$, upper) and ^{13}C (151 MHz, $CDCl_3$, lower) NMR spectra of **26DPN**.

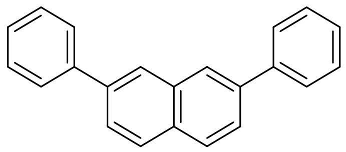

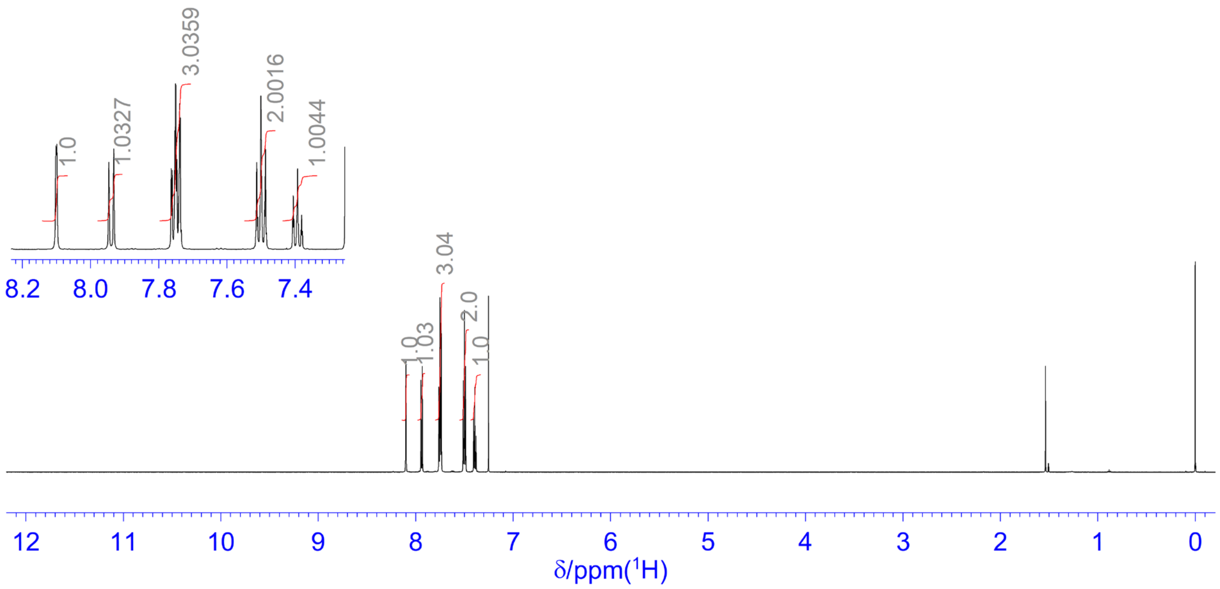

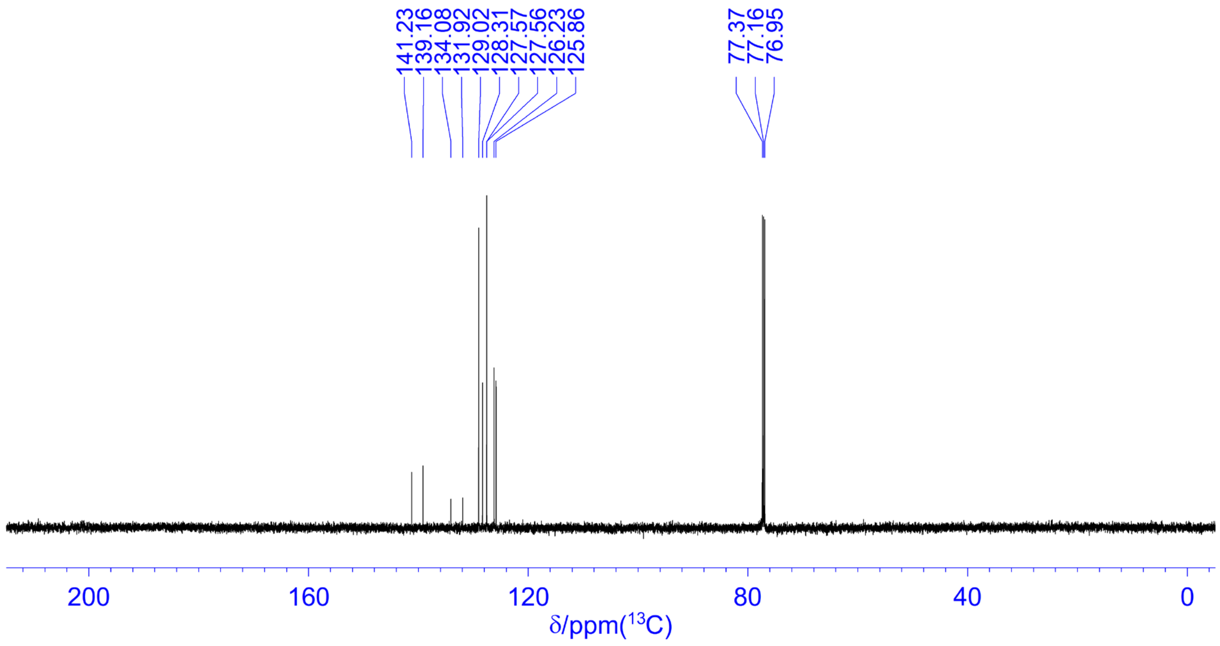

Figure S5. ^{1}H (600 MHz, $CDCl_3$, upper) and ^{13}C (151 MHz, $CDCl_3$, lower) NMR spectra of **27DPN**.

4. X-ray crystallographic analysis data of DPNs.

There have been reported crystallographic data of **14DPN** (CCDC ZAXJEP and 802922)[2]. The obtained crystal features and ORTEP are shown in Figures S6-9. Crystallographic data of the studied DPNs are listed in Tables S1-4. All non-hydrogen atoms were refined anisotropically; hydrogen atoms were refined isotropically, whereby hydrogen positions are based on stereochemical considerations. Pictures of the actually prepared crystals can be seen in Figure S10.

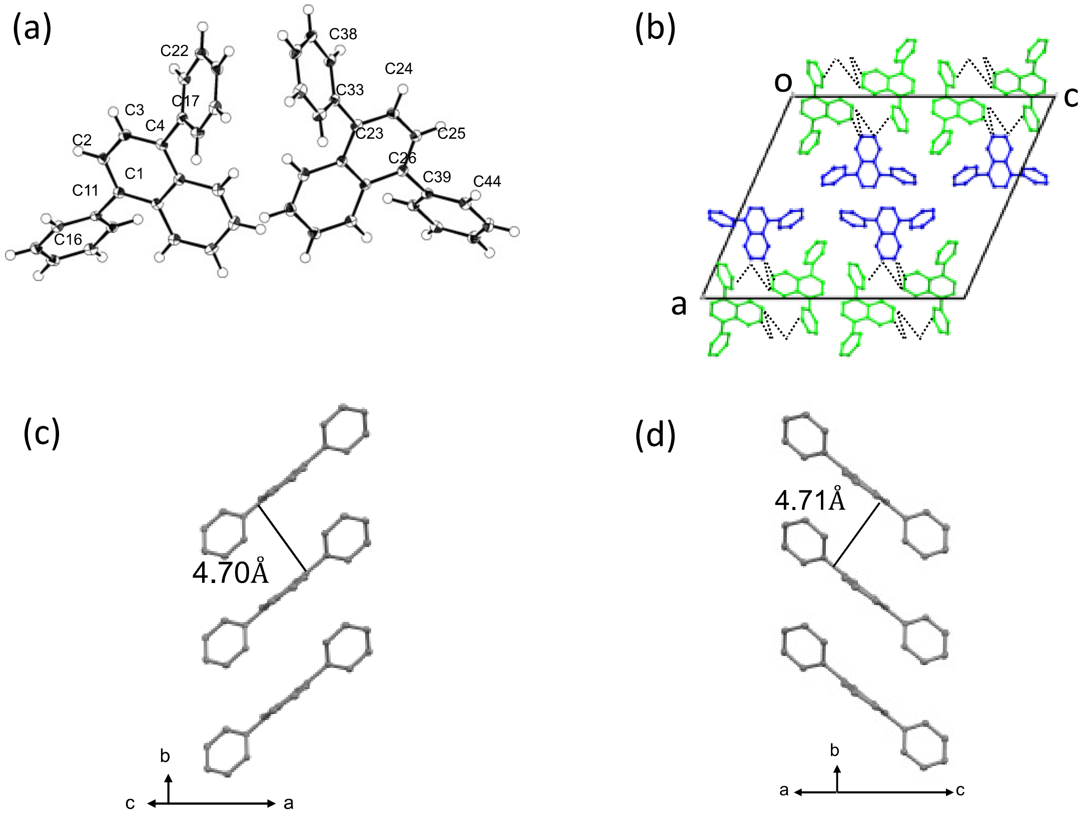

Figure S6. Crystal features of 14DPN. (a) ORTEP for the unit. (b) View along the b-axis. The molecule is colored by symmetry equivalence for C1-C22 (green) and C23-C44 (blue). The dotted lines note intermolecular CH-π bonds between the naphthalene moieties. (c) The molecular alley for C1-C22 (green). The distance between the naphthalene rings was 4.70 Å. (d) The molecular alley for C23-C44 (blue). The distance between the naphthalene rings was 4.71 Å.

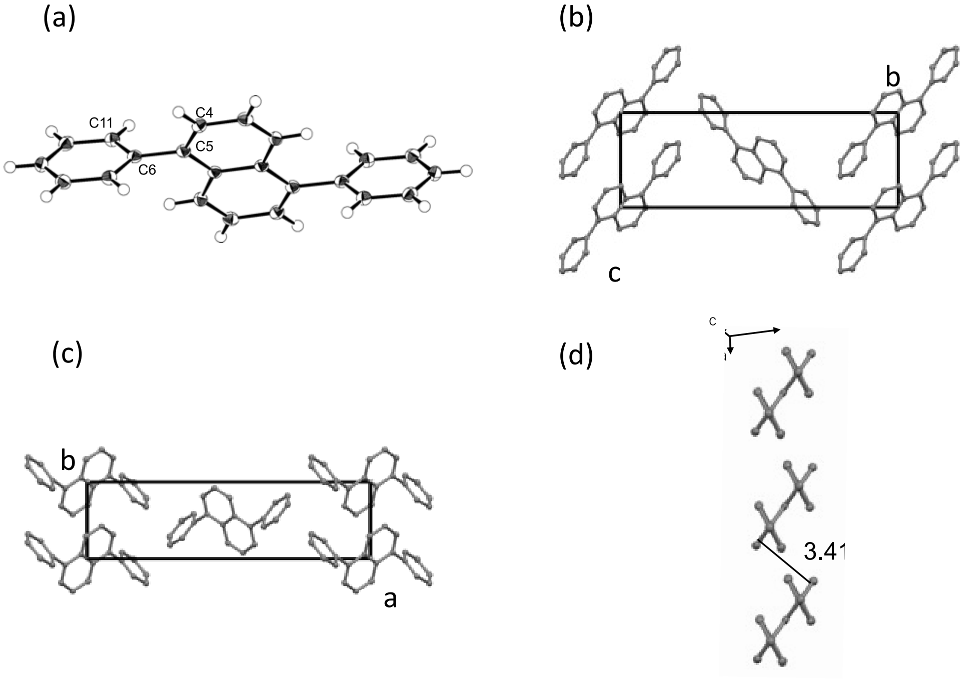

Figure S7. Crystal features of 15DPN. (a) ORTEP for the unit. (b) View along the a-axis. (c) View along the c-axis. (d) The molecular alley from the parallel direction to the naphthalene moiety. The distance between the naphthalene rings was 3.41 Å.

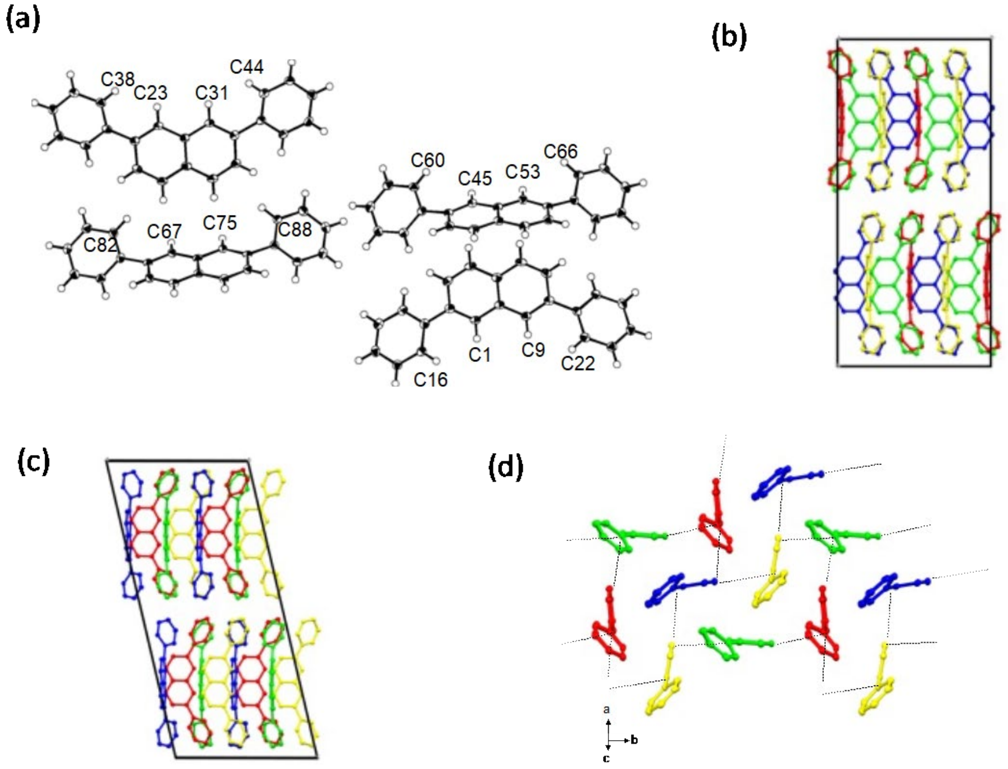

Figure S8. Crystal features of 27DPN-m. The molecule is colored by symmetry equivalence. (a) ORTEP for the unit. (b) View along the a-axis. (c) View along the b-axis. (d) View along the c-axis. The dotted lines note intermolecular CH-π bonds between the naphthalene moieties.

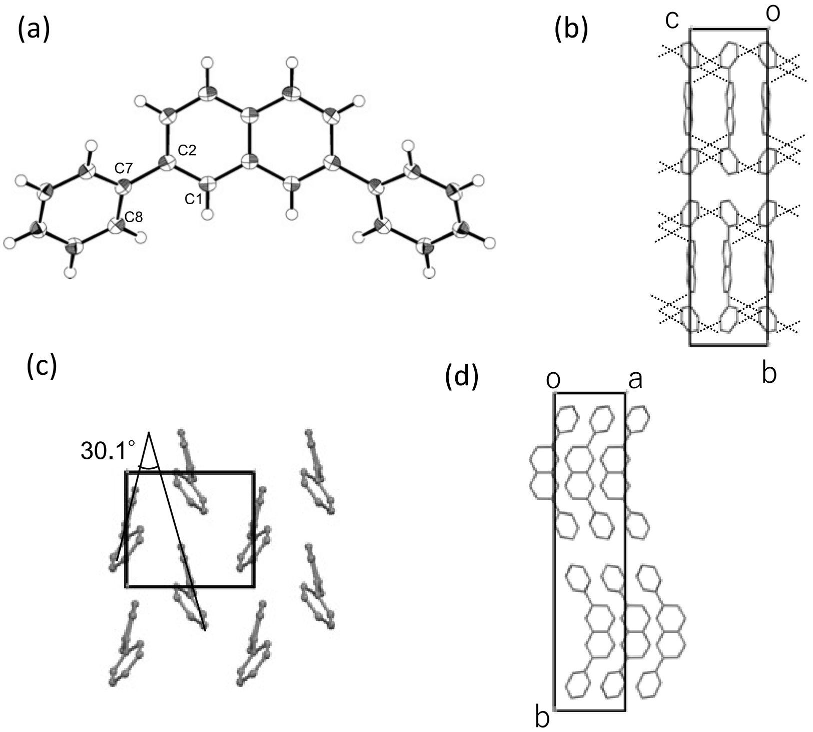

Figure S9. Crystal features of 27DPN-o. (a) ORTEP for the unit. (b) View along the a-axis. The dotted lines note intermolecular CH-π bonds between the naphthalene moieties. (c) View along the b-axis. The dihedral angle between the naphthalene rings was 30.1°. (d) View along the c-axis.

Figure S10. Pictures of the prepared crystals.

Table S1. Crystallographic data of compounds **14DPN** and**15DPN**

Compound	**14DPN**<br>CCDC 2176515	**15DPN**<br>CCDC 2176511
Empirical formula (FW)	$C_{22}H_{16}$ (280.35)	$C_{22}H_{16}$ (280.35)
Crystal dimensions	0.35 x 0.09 x 0.04 mm^3	0.30 x 0.08 x 0.03 mm^3
Crystal system	monoclinic	monoclinic
a	21.3809(4) Å	5.7768(2) Å
b	5.8909(1) Å	19.1732(5) Å
c	25.4471(6) Å	6.8593(2) Å
α	90 °	90 °
β	114.132(3) °	109.816(4) °
γ	90°	90 °
V	2925.01(11) Å^3	714.75(4) Å^3
Space group	$P2_1$ /c	$P2_1$ /n
Z	8	2
Z'	2	0.5
ρ_{calcd}	1.273 g/cm^3	1.303 g/cm^3
F(000)	1184	296
μ(CuKα)	0.544 mm^{-1}	0.556 mm^{-1}
$2\theta_{max}$	144.380 °	144.122 °
Obs. Temp.	93 K	93 K
Total reflections measured	Total: 16446<br>Unique: 5602<br>(R_{int} = 0.0284)	Total: 3044<br>Unique: 1338<br>(R_{int} = 0.0254)
Number of parameters	397	100
Data/parameter ratio	14.1	13.4
R_1 (I>2.00σ(I))	0.0509	0.0375
$wR2$<br>(All reflections)	0.1350	0.1039
GOF	1.166	1.079
Max/min residual density	0.304 / -0.253 e^- Å^3	0.210 / -0.205 e^- Å^3

Table S2. Crystallographic data of compounds **26DPN** and **27DPN**

Compound	**26DPN** CCDC 2176512	**27DPN-m** CCDC 2176513	**27DPN-o** CCDC 2176514
Empirical formula (FW)	$C_{22}H_{16}$ (280.35)	$C_{22}H_{16}$ (280.35)	$C_{22}H_{16}$ (280.35)
Crystal dimensions	0.05 x 0.03 x 0.02 mm^3	0.24 x 0.22 x 0.08 mm^3	0.30 x 0.15 x 0.06 mm^3
Crystal system	monoclinic	monoclinic	orthorhombic
a	15.4309(4) Å	14.1514(2) Å	6.63910(10) Å
b	7.3010(2) Å	14.0779(3) Å	29.9056(6) Å
c	6.4104(2) Å	30.6916(5) Å	7.34330(10) Å
α	90 °	90 °	90 °
β	90.570(3) °	103.2089(17) °	90 °
γ	90°	90 °	90 °
V	722.17(4) Å^3	5952.67(18) Å^3	1457.98(4) Å^3
Space group	$P2_1$ /c	Cc	Pnma
Z	2	16	4
Z'	0.5	4	0.5
ρ_{calcd}	1.289 g/cm^3	1.251 g/cm^3	1.277 g/cm^3
F(000)	296	2368	592
μ(CuKα)	0.551 mm^{-1}	0.534 mm^{-1}	0.545
$2\theta_{max}$	144.248 °	144.362 °	144.514 °
Obs. Temp.	93 K	93 K	93 K
Total reflections measured	Total: 3551 Unique: 1375 (R_{int} = 0.0194)	Total: 10211 Unique: 6133 (R_{int} = 0.0171)	Total: 13570 Unique: 1453 (R_{int} = 0.0379)
Number of parameters	100	794	103
Data/parameter ratio	13.8	7.72	14.1
R_1 (I>2.00σ(I))	0.0407	0.0326	0.0385
$wR2$ (All reflections)	0.1087	0.0887	0.1071
GOF	1.036	1.059	1.077
Max/min residual density	0.287 / -0.217 e$^-$ Å^3	0.156 / -0.237 e$^-$ Å^3	0.138 / -0.216 e$^-$ Å^3

Table S3. Torsion angles of the phenyl groups.

Compound	Carbon number	Torsion angle / deg	Z'
14DPN	C2 C1 C11 C16	-50.9(3)	2
	C3 C4 C17 C22	-50.9(3)	
	C24 C23 C33 C38	49.9(3)	
	C25 C26 C39 C44	54.2(3)	
15DPN	C4 C5 C6 C11	62.7(2)	0.5
26DPN	C1 C5 C6 C7	24.38(18)	0.5
27DPN-m	C1 C2 C11 C16	39.7(4)	4
	C9 C8 C17 C22	-43.2(4)	
	C23 C24 C33 C38	-36.3(4)	
	C31 C30 C39 C44	35.9(4)	
	C45 C46 C55 C60	-43.8(4)	
	C53 C52 C61 C66	42.7(4)	
	C67 C68 C77 C82	40.1(4)	
	C75 C74 C83 C88	-38.8(4)	
27DPN-o	C1 C2 C7 C8	26.5(1)	0.5

Table S4. Short contact between D and A found in Mercury software.

Compound	D-H···A	D-A / Å	H-A /Å	∠DHA / deg	D[a]	A[a]
14PNP	C9-H9···C31	3.551(3)	2.747	142.9	N	N
	C13-H13···C31	3.573(4)	2.879	130.9	P	N
	C30-H30···C8	3.697(3)	2.856	148.1	N	N
	C30-H30···C9	3.512(3)	2.894	123.8	N	N
	C32-H32···C15	3.491(4)	2.705	140.5	N	N
15PNP	none	-	-	-		
26PNP	C7-H4···C5	3.768(2)	2.868	158.52	P	N
	C7-H4···C6	3.595(2)	2.896	131.38	P	P
	C8-H5···C8	3.769(2)	2.866	159.18	P	P
	C11-H8···C11	3.772(2)	2.871	158.67	P	P
27PNP-m	C1 H1··· C70	3.800(4)	2.874	165.4	N	N
	C4-H3··· C45	3.773(4)	2.880	157.1	N	N
	C6-H4··· C53	3.719(4)	2.818	158.5	N	N
	C16-H11···C25	3.578(4)	2.762	144.5	P	N
	C22-H16···C29	3.572(4)	2.725	148.9	P	N
	C26-H19···C67	3.789(4)	2.852	169.2	N	N
	C28-H20···C75	3.719(4)	2.782	168.7	N	N
	C34-H23···C59	3.779(4)	2.877	159.0	P	P
	C34-H23···C58	3.512(4)	2.700	143.8	P	P
	C37-H26···C55	3.541(4)	2.854	130.2	P	P
	C37-H26···C60	3.584(4)	2.890	130.9	P	P
	C40-H28···C64	3.541(4)	2.854	130.1	P	P
	C40-H28···C65	3.667(4)	2.832	147.2	P	P
	C43-H31···C66	3.618(4)	2.854	138.2	P	P
	C43-H31···C61	3.557(4)	2.792	138.2	P	P
	C48-H35···C1	3.765(4)	2.874	156.5	N	N
	C53-H38···C28	3.806(4)	2.890	162.3	N	N
	C60-H43···C69	3.557(4)	2.701	150.1	P	P
	C66-H48···C73	3.581(4)	2.716	151.6	P	N
	C70-H51···C23	3.766(4)	2.831	168.2	N	N
	C72-H52···C31	3.811(4)	2.875	168.9	N	N
	C78-H55···C15	3.648(4)	2.853	141.9	P	P
	C81-H58···C11	3.550(4)	2.821	134.2	P	P
	C84-H60···C20	3.528(4)	2.732	141.9	P	P
	C85-H61···C58	3.623(4)	2.885	135.4	P	P
	C87-H63···C17	3.564(4)	2.895	128.5	P	P
	C87-H63···C22	3.591(4)	2.890	131.6	P	P
27PNP-o	C8-H4···C2	3.628(1)	2.802	145.86	P	N
	C9-H5···C8	3.654(1)	2.848	143.35	P	P
	C12-H8···C11	3.654(1)	2.844	143.77	P	P

a) N and P indicate the naphthalene and phenyl rings, respectively.

5. Decay profiles of fluorescence of the DPNs in cyclohexane, acetonitrile and the solid state.

Figure S11 shows decay profiles of fluorescence for PNs and DPNs in cyclohexane.

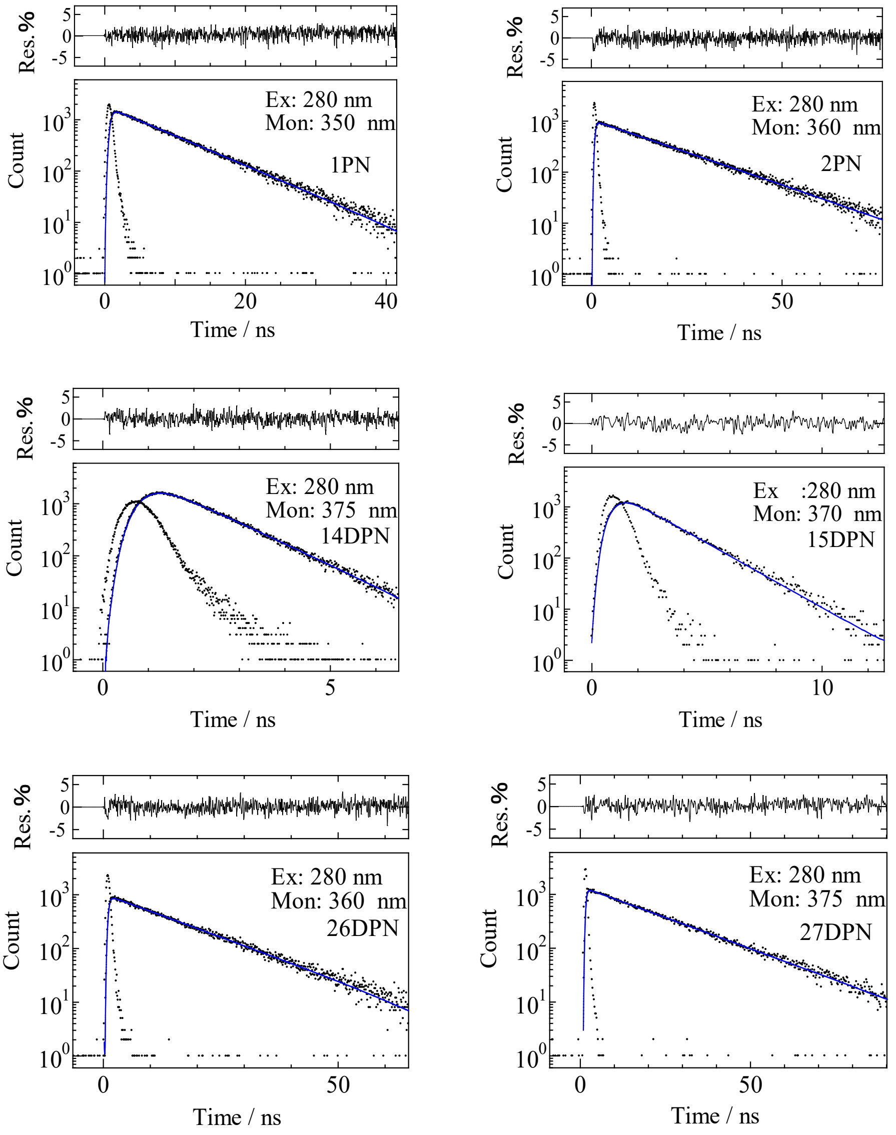

Figure S11. Decay profiles of fluorescence for PNs and DPNs in cyclohexane at 295 K. Ex and Mon in the figures indicate the excitation and monitoring wavelengths, respectively.

Figure S12 shows decay profiles of PNs and DPNs in acetonitrile.

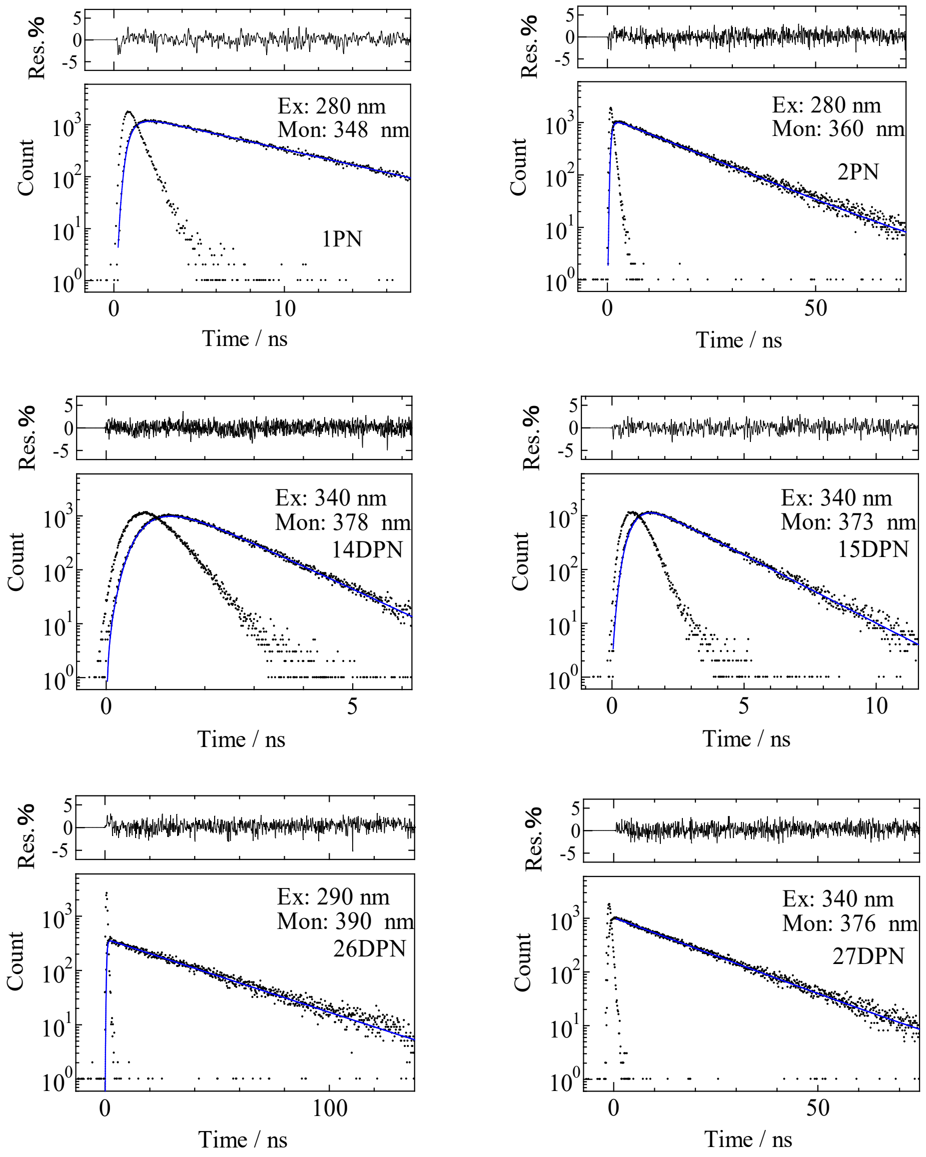

Figure S12. Decay profiles of fluorescence for PNs and DPNs in acetonitrile at 295 K. Ex and Mon in the figures indicate the excitation and monitoring wavelengths, respectively.

Figure S13 shows decay profiles for 2PN and DPNs in the solid state.

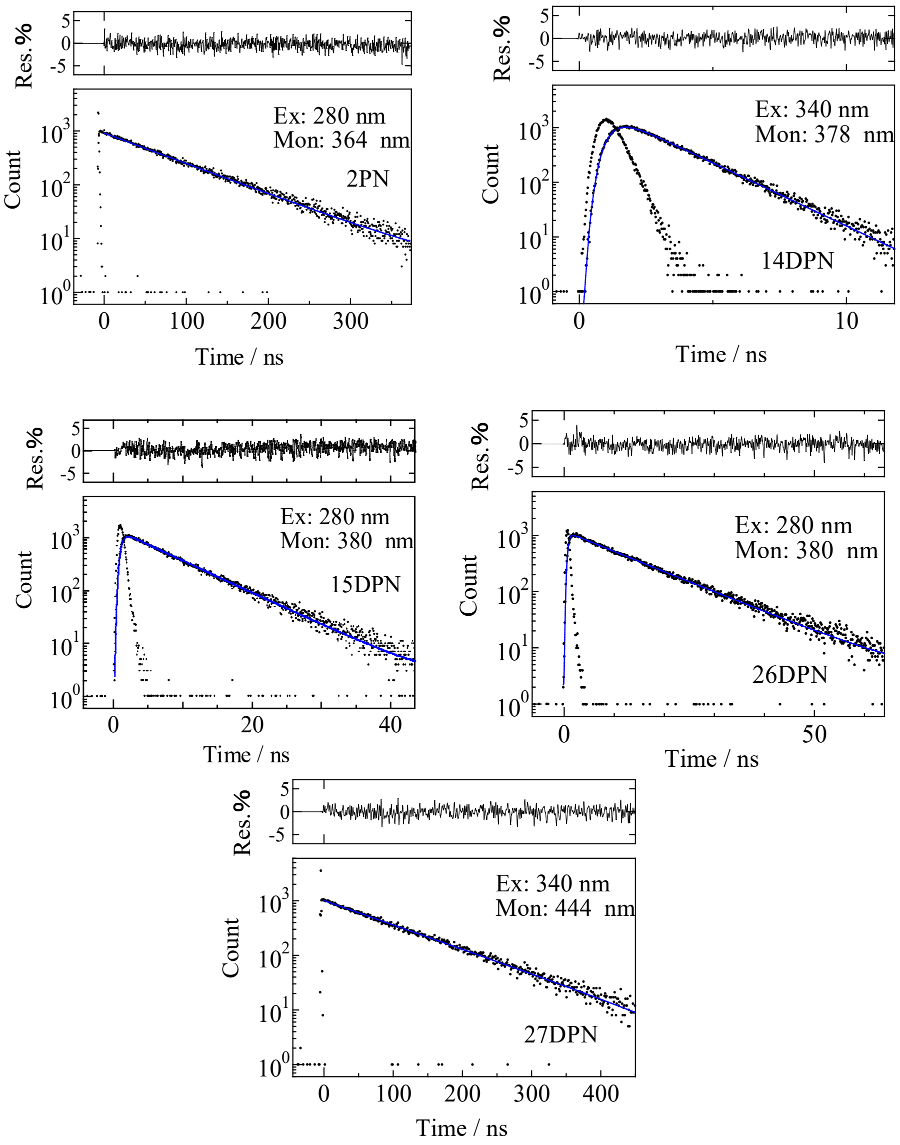

Figure S13. Decay profiles of fluorescence for 2PN and DPNs in the solid state at 295 K. Ex and Mon in the figures indicate the excitation and monitoring wavelengths, respectively.

6. Results of DFT and TD-DFT calculations of the DPNs; atom coordinates and sum of electronic and zero-point energies in cyclohexane.

The calculation was carried out at the DFT level, using the Gaussian 09 software package [3]. The geometries of DPNs were fully optimized by using the 6-31+G(d) base set at the B3LYP method. Atom coordinates for the optimized geometries of DPNs in cyclohexane are as follows.

Table S5. Atom coordinates for the optimized geometry of 14DPN.

Atom	X	Y	Z
C	-3.586024	-1.302010	-0.996308
C	-4.978842	-1.400883	-0.962650
C	-5.702256	-0.751871	0.039342
C	-5.027792	-0.016372	1.016246
C	-3.635039	0.075108	0.984626
C	5.037303	-0.019501	-1.011420
C	5.705015	-0.756902	-0.032390
C	4.975455	-1.395966	0.972668
C	3.583687	-1.290474	1.000883
C	2.907227	-0.539338	0.032003
C	-2.903436	-0.550517	-0.032336
C	3.644710	0.083509	-0.981840
C	-1.391042	2.024757	-0.172964
C	-0.711004	3.217746	-0.098369
C	0.692349	3.221285	0.102513
C	1.381122	2.033616	0.163892
C	1.417994	-0.460805	0.044696
C	0.712604	-1.645126	0.014233
C	-0.703765	-1.648914	-0.022728
C	-0.706649	0.776488	-0.049376
C	0.704222	0.779851	0.039198
C	-1.414796	-0.468826	-0.053156
H	-3.028495	-1.819413	-1.793890
H	-5.507784	-1.993538	-1.726685
H	-6.801160	-0.829981	0.067065
H	-5.594694	0.481299	1.819614
H	-3.114051	0.634138	1.779044
H	5.608727	0.469831	-1.816862

H	6.803127	-0.845679	-0.059235
H	5.499207	-1.988771	1.740026
H	3.020789	-1.804852	1.796866
H	3.129408	0.644761	-1.778337
H	-2.479130	2.061894	-0.338751
H	-1.254523	4.172485	-0.186304
H	1.228840	4.179695	0.197694
H	2.469817	2.076706	0.323539
H	1.244261	-2.610585	0.006767
H	-1.230604	-2.617109	-0.011129

Sum of electronic and zero-point energies = -847.880413607 Hartree

Table S6. Atom coordinates for the optimized geometry of 15DPN.

Atom	X	Y	Z
C	-4.144085	-0.775633	-0.882499
C	-5.484149	-0.409726	-0.737036
C	-5.869686	0.436230	0.304341
C	-4.912599	0.903169	1.207150
C	-3.575128	0.527964	1.064804
C	5.482618	0.404971	-0.737504
C	5.865940	-0.441360	0.304025
C	4.908504	-0.905573	1.208530
C	3.572183	-0.528765	1.065940
C	3.173652	0.299549	0.008664
C	-3.173833	-0.298945	0.007906
C	4.143513	0.775339	-0.881880
C	-0.905827	1.636087	-0.257203
C	0.137683	2.528995	-0.270615
C	1.474825	2.064694	-0.188623
C	1.749761	0.715359	-0.130304
C	0.909238	-1.633077	-0.256114
C	-0.133824	-2.526024	-0.273095
C	-1.471274	-2.061923	-0.188419
C	-0.667241	0.230007	-0.166218
C	0.668684	-0.227141	-0.165242

C	-1.748668	-0.712881	-0.130887
H	-3.857803	-1.442764	-1.711605
H	-6.239475	-0.789820	-1.444016
H	-6.927005	0.724733	0.420544
H	-5.215671	1.557546	2.040533
H	-2.837975	0.879699	1.804780
H	6.238102	0.782768	-1.445495
H	6.922419	-0.732606	0.419710
H	5.210191	-1.559573	2.042705
H	2.833554	-0.879898	1.804876
H	3.857825	1.444048	-1.710044
H	-1.928265	2.036222	-0.339004
H	-0.060708	3.611410	-0.334106
H	2.290498	2.805262	-0.154136
H	1.931943	-2.033076	-0.336511
H	0.066196	-3.607981	-0.340649
H	-2.285548	-2.803857	-0.151917

Sum of electronic and zero-point energies = -847.880409158 Hartree

Table S7. Atom coordinates for the optimized geometry of 26DPN.

Atom	X	Y	Z
C	4.839597	-1.114360	0.438967
C	6.227586	-0.965317	0.495662
C	6.825309	0.227852	0.078486
C	6.024979	1.269347	-0.397706
C	4.636426	1.119491	-0.459228
C	-4.869307	1.115335	-0.408126
C	-6.253829	0.940674	-0.459377
C	-6.827945	-0.271080	-0.065060
C	-6.003429	-1.306590	0.383711
C	-4.619175	-1.131680	0.436341
C	4.024740	-0.073503	-0.038529
C	-4.025392	0.080416	0.035897
C	-0.616193	1.683450	0.562118
C	-1.979848	1.503765	0.507661

C	-2.546000	0.260459	0.092575
C	-1.683205	-0.766794	-0.261698
C	0.619235	-1.660229	-0.573775
C	1.984680	-1.481112	-0.517551
C	2.544686	-0.240083	-0.098044
C	0.280649	0.640692	0.204452
C	-0.273172	-0.614731	-0.214351
C	1.690206	0.792941	0.253813
H	4.377553	-2.030629	0.793752
H	6.838178	-1.780757	0.872804
H	7.904368	0.343234	0.120767
H	6.477983	2.198293	-0.732581
H	4.020437	1.918794	-0.859038
H	-4.433477	2.055922	-0.728799
H	-6.883189	1.752940	-0.811838
H	-7.904670	-0.405449	-0.103474
H	-6.437389	-2.250187	0.702382
H	-3.986606	-1.931819	0.807179
H	-0.206228	2.635488	0.890304
H	-2.642917	2.310668	0.800681
H	-2.090731	-1.713552	-0.605746
H	0.209278	-2.611320	-0.904125
H	2.651742	-2.280699	-0.823735
H	2.102083	1.735410	0.604032

Sum of electronic and zero-point energies = -847.893940039 Hartree

Table S8. Atom coordinates for the optimized geometry of 27DPN.

Atom	X	Y	Z
C	-4.993515	0.401840	0.016477
C	-6.194749	-0.288478	0.020300
C	-6.190526	-1.680555	0.006672
C	-4.965081	-2.344008	-0.010794
C	-3.769387	-1.622324	-0.017012
C	-3.748930	-0.248812	-0.004030
C	1.247036	-0.178257	-0.004631

C	2.462140	0.468960	0.001540
C	2.425717	1.894106	0.004828
C	1.250718	2.599784	-0.001594
C	-1.216792	2.587381	-0.000305
C	-2.407315	1.893596	-0.008897
C	-2.458108	0.489539	-0.009373
C	-0.003601	0.508264	-0.007072
C	0.001066	1.922531	-0.007089
C	-1.243820	-0.174133	-0.006511
C	3.738009	-0.261565	0.003813
C	3.779400	-1.659976	0.008985
C	4.978618	-2.360514	0.007098
C	6.183712	-1.670671	0.001823
C	6.153069	-0.278803	-0.000631
C	4.968269	0.413763	-0.000166
H	-5.070832	1.498141	0.031274
H	-7.146625	0.261247	0.035406
H	-7.132957	-2.244366	0.010256
H	-4.941935	-3.443096	-0.020619
H	-2.850673	-2.225866	-0.033319
H	1.190329	-1.276341	-0.008614
H	3.350461	2.488449	0.012738
H	1.273199	3.699259	-0.000212
H	-1.225107	3.687080	0.001732
H	-3.321449	2.503681	-0.004409
H	-1.198639	-1.272652	-0.000193
H	2.863359	-2.267528	0.014139
H	4.973544	-3.459916	0.009618
H	7.138872	-2.212689	-0.000208
H	7.098124	0.283121	-0.004086
H	5.024755	1.511461	-0.004092

Sum of electronic and zero-point energies = -847.886818816 Hartree

References

1. Ho, J.-H.; Chen, Y.-H.; Chou, L.-T.; Lai, P.-W.; Chen, P.-S. The improvement of π-conjugation by the lateral benzene of anthracene and naphthalene. *Tetrahedron Lett.* **2014**, *55*, 5727-5731, doi:10.1016/j.tetlet.2014.08.097.
2. Lima, C.F.; Rocha, M.A.; Schroder, B.; Gomes, L.R.; Low, J.N.; Santos, L.M. Phenylnaphthalenes: sublimation equilibrium, conjugation, and aromatic interactions. *J. Phys. Chem. B* **2012**, *116*, 3557-3570, doi:10.1021/jp2111378.
3. Frisch, M.J.; Trucks, G.W.; Schlegel, H.B.; G. E. Scuseria; Robb, M.A.; Cheeseman, J.R.; Scalmani, G.; Barone, V.; Mennucci, B.; Petersson, G.A.; et al. *Gaussian 09, Revision D.01*, Gaussian, Inc.: Wallingford CT, 2010.
